# Effect of fatty acid-enriched black soldier fly larvae meal combined with chitinase on the metabolic processes of Nile tilapia

**DOI:** 10.1017/S0007114523003008

**Published:** 2024-04-28

**Authors:** Pamphile S. Agbohessou, Robert Mandiki, Wouter Mes, Aude Blanquer, Mazarine Gérardy, Mutien-Marie Garigliany, Jérôme Lambert, Pierre Cambier, Nicole Tokpon, Philippe A. Lalèyè, Patrick Kestemont

**Affiliations:** 1 Research Unit in Environmental and Evolutionary Biology (URBE), Institute of Life, Earth and Environment (ILEE), University of Namur, Namur, Belgium; 2 Laboratory of Hydrobiology and Aquaculture (LHA), Faculty of Agronomics Sciences (FSA), University of Abomey-Calavi (UAC), Cotonou, Benin; 3 Department of Microbiology, Radboud Institute for Biological and Ecological Sciences (RIBES), Radboud University, Nijmegen, the Netherlands; 4 Department of Veterinary Pathology, FARAH, Faculty of Veterinary Medicine, University of Liège, 4000, Liège, Belgium; 5 Unit of Research in Plant Cellular and Molecular Biology, Institute of Life, Earth and Environment (ILEE), University of Namur, Namur, Belgium

**Keywords:** *Oreochromis niloticus*, *Hermetia illucens*, Chitinase, Growth, Gut microbiota

## Abstract

The aim of this study is to determine to what extent the addition of chitinase to black soldier fly (BSF) larval meal enriched or not with long-chain PUFA (LC-PUFA) could improve growth, protein digestion processes and gut microbial composition in Nile tilapia. Two different types of BSF meal were produced, in which larvae were reared on substrates formulated with vegetable culture substrate (VGS) or marine fish offal substrate (FOS). The BSF raised on VGS was enriched in *α*-linolenic acid (ALA), while that raised on FOS was enriched in ALA + EPA + DHA. Six BSF-based diets, enriched or not with chitinase, were formulated and compared with a control diet based on fishmeal and fish oil (FMFO). Two doses (D) of chitinase from *Aspergillus niger* (2 g and 5 g/kg feed) were added to the BSF larval diets (VGD0 and FOD0) to obtain four additional diets: VGD2, VGD5, FOD2 and FOD5. After 53 d of feeding, results showed that the BSF/FOS-based diets induced feed utilisation, protein efficiency and digestibility, as well as growth comparable to the FMFO control diet, but better than the BSF/VGS-based diets. The supplementation of chitinase to BSF/FOS increased in fish intestine the relative abundance of beneficial microbiota such as those of the *Bacillaceae* family. The results showed that LC-PUFA-enriched BSF meal associated with chitinase could be used as an effective alternative to fishmeal in order to improve protein digestion processes, beneficial microbiota and ultimately fish growth rate.

Recently, protein meals derived from insects have attracted attention as a new feed ingredient for aquaculture feeds due to their potential beneficial properties in terms of growth and health^(1)^. Among insect species, the black soldier fly (BSF, *Hermetia illucens* (L. 1758)) is very interesting because these larvae have the ability to convert food waste into high-quality proteins^(2)^. BSF larvae meals contain between 30 and 58 % crude protein with a balanced profile of essential amino acids close to that of fishmeal (FM), as well as appropriate levels of minerals and vitamins^(3)^. A meta-analysis showed that inclusion of BSF meal at generally moderate levels did not affect fish growth, while incorporation at high levels ≥ 29 % could reduce growth compared to FM^(4)^. For example, a partial replacement of 70 % of FM by BSF larvae meal did not affect the growth of Nile tilapia (*Oreochromis niloticus* (L. 1758))^(5)^, while our previous study showed that total replacement of FM with BSF larvae meal reduced the growth in the same fish species^(6)^.

BSF larvae meal contains SFA but is poor in PUFA and long-chain PUFA (LC-PUFA)^(7)^. The lack of PUFA or LC-PUFA in BSF larval meal limits their feeding efficiency in fish, especially when FM and fish oil (FO) are replaced by plant ingredients^(6)^. The PUFA deficiencies in fish diets can decrease feed intake (FI) and reduce growth and even increase mortality. Different approaches have been investigated to enrich BSF meal before incorporation into fish diets. For example, BSF larvae fed with fish offal or marine algae were enriched in EPA (C20:5) and DHA (C22:6)^(8)^. In our previous study, none of the *α*-linolenic acid (ALA)- and EPA-enriched insect meals improved the protein efficiency ratio (PER) or protein digestibility, but decreased growth was observed in Nile tilapia^(6)^. Similarly, protein and amino acid digestibility was reduced in Atlantic salmon when they were fed diets containing 60 % BSF larval meal compared with a FM diet^(9)^. These authors attributed the low digestibility to the presence of chitin (5·6–6·7 %) in BSF larval meals because chitin can interfere with intestinal homoeostasis^(10)^. Feed supplemented with chitin had lowered nutrient digestibility, weight gain and feed conversion in *O. niloticus × O. aureus* ^(11)^. Fish that eat insects increase the activity of enzymes such as chitinase and chitobiase to facilitate digestion^(12)^. A significant increase in fish-specific growth was observed with dietary supplementation of 10 and 20 mg/kg feed of recombinant grouper (*Epinephelus coioides*) chitinase1 (rgChi1) protein in a diet containing 2 % shrimp shell chitin^(13)^. The structure of gut microbial communities, including microbial diversity, is strongly influenced by diet ingredients, as the microbiota responds rapidly to dietary changes^(14)^. In addition, the bioactive compounds present in insects can modify the complex communities of the intestinal microbiota of the fish that consume them. Therefore, BSF-based diets can modify the biodiversity of the fish microbiome community structure; however, information on the BSF actions on fish gut microbiota remains limited. It has been shown that full-fat BSF diet induced a differential response in Atlantic salmon presmolts resulting in gut microbiota composition dominated by beneficial lactic acid bacteria and Actinomyces and showed a predicted increase in mucin degradation compared with the other diets^(15)^. Moreover, the consumption of BSF modulated the diversity and richness of bacteria involved in the process of nutrition and immunity of rainbow trout (*O. mykiss*)^(16)^. The results of ref. 17 revealed that *Actinobacteria* and *Firmicutes* were most abundant in the gut of BSF and fish fed BSF diets, while *Proteobacteria* were the dominant bacteria in the gut of fish fed FM. The gut microbiota modulation can also affect lipid metabolism and the balance of energy^(18)^. It also seems that certain BSF diets do not improve the microbiota diversity in carnivorous fish in a balanced way but could be more beneficial in herbivore and detritivore fish species^(19)^.

The present study aimed to determine if the addition of chitinase to BSF meals enriched or not in LC-PUFA could improve growth-related parameters and gut microbiota status in Nile tilapia. To achieve this objective, growth, feed utilisation, PER, digestibility, digestive enzymes, expression of target genes involved in the processes of digestive enzymes and lipid metabolism, and gut microbiota composition were measured in Nile tilapia juveniles fed diets based on BSF meals enriched in PUFA/LC-PUFA with increasing doses of added chitinase.

## Materials and methods

### Enrichment of black soldier fly larvae meals

The BSF larval cultures and the tests of fish feeding were carried out at the application farm of the Faculty of Agronomics Sciences of the University of Abomey-Calavi (FSA/UAC) in Benin. Samples were transferred to the Research Unit in Environmental and Evolutionary Biology, Namur University, Belgium, for biochemical, gene expression and gut microbiota analyses. Feeding trial was carried out in agreement with the European and Belgian national legislation on fish welfare (protocol number: KE20/350).

The methodology of PUFA and LC-PUFA production and enrichment in BSF larvae was reported in our previous study^(6)^. In summary, we formulated two kinds of substrates to rear BSF larvae. A vegetable culture substrate (VGS) composed of soyabean, seeds and leaves of *Euphorbia heterophylla*, and rapeseed oil to enrich BSF larvae (BSF/VGS) with linoleic acid (LA) and ALA. A fish offal substrate (FOS) composed of soyabean meal and marine fish offal to enrich BSF larvae (BSF/FOS) with EPA and DHA. The chemical composition of the BSF larval meals produced is shown in Table 1.

**Table 1.** Analysed chemical composition of black soldier fly (BSF) larvae produced on different substrates: fish offal substrate (FOS) and vegetable culture substrate (VGS)

Chemical composition	BSF/FOS	BSF/VGS
DM (g/kg)	358·08	360·76
Ash (g/kg DM)	76·25	68·90
Crude fat (g/kg DM)	267·10	329·20
Crude protein (g/kg DM)	450·80	416·00
Main fatty acid composition (% of total identiﬁed fatty acids)		
C12:0	32·00	38·94
C14:0	6·37	5·93
C16:0	16·85	7·90
C18:0	3·48	1·40
Total SFA	60·82	55·60
C16:1	0·59	2·60
C17:1	0·88	0·27
C18:1n9	17·67	22·13
C20:1n9	00	0·64
Total monoenes	19·42	25·56
C18:2n6 (LA)	6·19	10·08
Total *n*-6 PUFA	6·22	10·34
C18:3n3 (ALA)	0·52	2·30
C20:3n3	0·91	00
C20:5n3 (EPA)	2·20	00
C22:6n3 (DHA)	0·39	00
Total *n*-3 PUFA	4·02	2·30
* n*-3:*n*-6	0·64	0·22

LA, linoleic acid; ALA, *α*-linolenic acid.

### Facilities and fish

#### Experimental diets

Seven iso-nitrogenous (crude protein = 31·8 %) and isolipid (crude lipids = 12·3–12·9 %) diets were formulated (Tables 2 and 3). A control diet containing fishmeal and fish oil (FMFO) was formulated. Two diets VGD0 and FOD0 were formulated with each type of BSF meal to completely replace FM, and FO was replaced with palm oil. We supplemented two doses (2 g/kg DM and 5 g/kg DM of feed) of commercial chitinase from *Aspergillus niger* (Creative Enzymes) to the diets that contain BSF/FOS and BSF/VGS meals to formulate the FOD2, FOD5, VGD2 and VGD5 diets. The composition of the FOD diets formulated with BSF meal from larvae reared on FOS substrate were all enriched in EPA and DHA, while the VGD diets formulated with BSF meal from larvae reared on VGS substrate were all enriched in ALA. The plant ingredients were powdered and then cooked over heat, and the different doses of chitinase were added, and then all the feed ingredients were homogenised. Water was gradually added, and the mixture passed through a semi-extruder (BD-GP70, Henan BEDO Machinery Equipment) to produce 2 mm granules. The pellets were dried at room temperature until they contained no more water, before being stored at −4°C. The fish were therefore fed the seven experimental diets manufactured (2 × 3 factorial design, with three levels of chitinase and two different meals of BSF larvae).

**Table 2.** Composition of experimental diets

Diets	FMFO	FOD0	FOD2	FOD5	VGD0	VGD2	VGD5
BSF prepupal meal	0	217·2	217·2	217·2	225·6	225·6	225·6
Fishmeal	110	0	0	0	0	0	0
Soyabean meal	233·1	242·4	243·4	246·4	242·5	242·5	245·5
Maize flour	430·5	350·3	347·3	341·3	341·8	339·8	333·8
Betaine	20	20	20	20	20	20	20
Menhaden fish oil	30	0	0	0	0	0	0
Palm oil	48	38·7	38·7	38·7	38·7	38·7	38·7
Blood meal	100	100	100	100	100	100	100
Carboxymethyl cellulose	5	5	5	5	5	5	5
Mineral premix	10	10	10	10	10	10	10
Vitamin premix	10	10	10	10	10	10	10
BHT	0·2	0·2	0·2	0·2	0·2	0·2	0·2
BHA	0·2	0·2	0·2	0·2	0·2	0·2	0·2
Methionine	3	5	5	5	5	5	5
Tryptophan	0	1	1	1	1	1	1
Chitinase	0	0	2	5	0	2	5
Total	1000	1000	1000	1000	1000	1000	1000
DM (g/kg)	908·54	912·49	910·04	909·74	911·44	909·65	908·77
Ash (g/kg DM)	57·62	58·44	61·98	71·06	55·81	57·56	51·65
Lipid (g/kg DM)	123	127·4	127·4	127·4	129·7	129·7	129·7
Protein (g/kg DM)	318·4	318·2	318·5	318·8	318·8	318·7	318·6
Chitin (g/kg DM)	0	10·9	10·9	10·9	11·3	11·3	11·3
Hexosamine (%)	3·17	3·57	3·47	3·52	3·27	3·63	4·16

FMFO, control diet with fishmeal (FM) and fish oil (FO); FOD, diet based on BSF larvae meal obtained from larvae grown on fish offal substrate; VGD, diet based on BSF larvae meal grown on a vegetable culture substrate; BSF, black soldier fly; BHA, butylated hydroxyanisole; BHT, butylated hydroxyl toluene.

The number added to the name of the diet indicates the dose of chitinase (2 or 5 g/kg of feed).

The source of the ingredients used to manufacture the diets has already been described in ref. 22.

**Table 3.** Composition of fatty acids (% of total fatty acids identiﬁed) in experimental diets

Main fatty acid	FMFO	FOD0	FOD2	FOD5	VGD0	VGD2	VGD5
C12:0	0·16	29	29	29	33·71	33·71	33·71
C14:0	2·95	5·85	5·85	5·85	5·22	5·22	5·22
C16:0	31·52	22·68	22·68	22·68	13·82	13·82	13·82
C18:0	4·26	0·59	0·59	0·59	2	2	2
Total SFA	38·89	58·12	58·12	58·12	54·75	54·75	54·75
C16:1*n*-7	3·39	0·59	0·59	0·59	2·29	2·29	2·29
C18:1*n*-9	30·37	23·95	23·95	23·95	26·45	26·45	26·45
C20:1*n*-9	1·17	0	0	0	0·54	0·54	0·54
Total monoenes	34·93	24·54	24·54	24·54	29·28	29·28	29·28
C18:2*n*-6 (LA)	17·1	11·02	11·02	11·02	13·65	13·65	13·65
C20:4*n*-6 (ARA)	0·15	0	0	0	0	0	0
Total *n*-6 PUFA	17·25	11·02	11·02	11·02	13·65	13·65	13·65
C18:3*n*-3 (ALA)	0·82	0·68	0·68	0·68	2·26	2·26	2·26
C20:5*n*-3 (EPA)	4·64	2	2	2	0	0	0
C22:6*n*-3 (DHA)	3·23	0·1	0·1	0·1	0	0	0
Total *n*-3 PUFA	8·69	2·78	2·78	2·78	2·26	2·26	2·26
*n*-3:*n*-6	0·5	0·25	0·25	0·25	0·17	0·17	0·17

FMFO, control diet with fishmeal (FM) and fish oil (FO); FOD, diet based on black soldier fly larvae meal obtained from larvae grown on fish offal substrate; VGD, diet based on black soldier fly larvae meal grown on a vegetable culture substrate; LA, linoleic acid; ALA, *α*-linolenic acid; ARA, arachidonic acid.

#### Experimental design

Juvenile male sex-reversed Nile tilapia were produced and then acclimated to experimental conditions for 3 weeks in fibreglass tanks installed in a recirculating aquaculture system with mechanical and biological water filtration systems. The fish were hand-fed until apparent satiety three times a day at 09.00, 13.00 and 17.00 with a commercial tilapia feed (BIOMAR).

A total of 630 fish with an average initial weight of 5·57 ± 0·05 g were randomly placed in twenty-one glass tanks of 100 litres with thirty fish per tank (three tanks per experimental diet). Each tank was supplied with 3–4 litres/min of water by gravity at a temperature of 31·1 ± 1·7°C and dissolved oxygen of 5·48 ± 0·31 mg/l. Nitrite and ammonia levels were checked weekly and reached a mean of 0·09 ± 0·05 mg/l and 0·08 ± 0·004 mg/l, respectively.

#### Feed utilisation and growth performance

All fish were individually weighed at the beginning and end of the experiment to determine growth performance. Daily FI was recorded to determine feed utilisation. The mortality was recorded daily. Growth performance and feed utilisation were determined using the following formulas:

Survival (%) = 100 × (final number of survivors per tank/initial number per tank)

Specific growth rate (SGR, %/day) = [ln (fw)-ln (iw)]/Δt × 100

Feed intake (FI) = FC/(N × Δt)

Feed efficiency (FE) = wet weight gain per fish (g)/feed consumed per fish (g)

Protein efficiency ratio (PER) = weight gain (g)/protein consumed (g)

where iw and fw are initial and final weights (g), Δt is duration of a trial, FC is feed consumption per tank (g), and N is number of surviving fish per tank.

#### Sample collection and biochemical analysis

On day 53, at the end of the feeding trial, six fish were randomly collected from each tank and anaesthetised with MS222 (anesthesia tricaine methanesulfonate) (Sigma-Aldrich) at a dose of 120 mg/l. The stomach and distal intestine (from the spiral part of the intestine to the anus) were removed and snap-frozen in liquid N_2_ after dissection and stored at –80°C until analysis to determine digestive enzyme activity. The middle intestine (the spiral part of the gut) was aseptically removed from the abdominal cavity, placed in a sterile 1·5 ml Eppendorf tube, transferred to liquid N_2_, and stored at –80°C until analysis of the expression of some genes involved in digestive enzyme and lipid metabolism processes and the gut microbiota.

The proximal composition (moisture, ash, protein and fat) of BSF larval meals (Table 1) and experimental diets (Table 2) used in the trial were analysed in the laboratory according to standard protocols described in ref. 6. To determine the moisture level, the samples were dried in an oven at 105°C for 12 h. Ash was analysed by incineration at 550°C in a muffle furnace for 5 h. Crude protein content was estimated (total nitrogen × 6·25 for feed and total nitrogen × 4·76 for BSF larvae meal) using the Kjeldahl distillation method after digestion of samples with sulphuric acid^(20,21)^. Lipids were extracted with chloroform/methanol (2:1, v:v) using the Folch method^(22)^. The fatty acid (FA) composition of the feed was estimated by GC^(6)^ (Table 3).

#### Digestive enzyme activities

From three fish per tank (nine fish per treatment), stomach and gut samples were homogenised in a solution of ten volumes (v/w) of PBS (NaCl: 6 mM, NaH_2_PO4:20 mM, pH 6·9). Intestinal alkaline phosphatase activity was measured using 4-nitrophenyl phosphate as substrate in 30 mM Na_2_CO_3_ buffer (pH 9·8)^(23)^. Intestinal aminopeptidase activity is measured using 80 mM sodium phosphate buffer (pH 7·0) and L-leucine p-nitroanilide as substrate (in 0·1 mM dimethylsulfoxyde)^(24)^. Intestinal trypsin activity was measured using BAPNA (N-*α*-benzoyl-dl-arginine p-nitroanilide) as substrate in 50 mM TRIS-HCl, 20 mM CaCl_2_, pH 8·2 buffer^(25)^. Intestinal amylase activity was measured using 0·3 % soluble starch dissolved in Na_2_HPO_4_ buffer pH 7·4 as substrate^(26)^. Stomach pepsin activity was measured using 2 % Hb in 1N HCl buffer as substrate^(27)^. The measure of chitinase activity in the stomach was performed using p-nitrophenyl (Sigma-Aldrich) as a substrate according to the method described by^(28)^, with a slight modification. Briefly, a mixture of 19·5 ul of a 0·2 M phosphate + 0·1 M citrate buffer solution (pH 6·0) and 7·5 μl of substrate solution was added to 7·5 μl of the gut homogenate. The mixture was incubated at 37°C for 20 min, then 200 μl of a 0·2 M sodium carbonate solution was added to the mixture and the absorbance of p-nitrophenol release was measured at 420 nm. The amount of enzyme releasing 1 μmol of p-nitrophenol per minute was defined as the chitinolytic enzyme activity unit (U). Protein concentration was determined according to the method of ref. 29. The unit of measurement for enzyme activities is the U or mU/mg of protein.

#### Gene expression analyses

Analyses of gene expression by quantitative real-time PCR (qPCR) were performed as described previously^(30)^. The total RNA of the middle intestine was isolated individually from three fish per tank (nine fish per treatment) according to the manufacturer’s instructions. To determine gene expression levels, the qPCR efficiency of each gene was tested before analysing. As a housekeeping gene, *18S* was chosen as it exhibited the most stable expression between samples (compared with *β*-*actin*). The expressions of enzymatic genes such as *chid1* (chitinase domain containing 1), *endochitinase A*, *ctbs* (chitobiase), *pept2* (solute carrier family 15 member 2), *tryp* (trypsinogen), *a*-*amy* (*α*-amylase), *apoa1* (apo A-1), *sglt1* (sodium/glucose cotransporter 1), *muc2* (mucin-2) and those of lipid metabolism such as *fads2* (FA desaturase 2), *fads6* (FA desaturase delta 6) and *elovl5* (very long elongase delta 5) were determined in the intestine tissues. Specific primers were designed on Primer3 software, and their quality was double-checked on Amplifix software with reference to tilapia sequences published on Genbank (Table 4). For each sample, the relative expression of the target or reference genes was obtained by the standard curve method previously described in ref. 30. The values for each sample were then expressed as normalised relative expression (NRE), determined by the formula NRE = relative concentration of target gene/relative concentration of reference gene. The average of the values of two fish per tank was calculated and considered as the experimental unit.

**Table 4.** Primer sequences used for the analysis of the expression of certain genes related to Nile tilapia

Gene	Protein	Primer sequence (5′-3′)	Reference
Enzymatic genes			
*chid1*	Chitinase domain containing 1	Fw:5′-TTACGGGACTCCACGATCAC-3′	XM_003440467·5
		Rv:5′-TTTGCAACATCCACCAGCTC-3′	
*endochitinase A*	Endochitinase A	Fw:5′-GGCCATCTACCACATCTACC-3′	XM_025898547·1
		Rv:5′-CTATTGGTGCAGCAGGAAGAC-3′	
*ctbs*	Chitobiase	Fw:5′-TCTCCGTCAGACACTCGATG-3′	XM_003452536·5
		Rv:5′-TGACATCGAGCTGTTGACCT-3′	
*pept2*	Solute carrier family 15 member 2	Fw:5′-AAAACGAAGACGGGAATCCA-3′	*XM_005475385·4*
		Rv:5′-AGGTTAAGTTTAGAGGTCTTCCCT-3′	
*tryp*	Trypsinogen	Fw:5′-AGTGCGCAAAGAACTCTGTG-3′	AY510093·1
		Rv:5′-AATGTTGTGCTCACCAAGGC-3′	
*a*-*amy*	*α*-Amylase	Fw:5′-TGGCGTTGGGCTGACATTGC-3′	ENSONIG00000018530
		Rv:5′-TTCCTGTTCCACCACCAGATCC-3′	
*apoa1*	Apo A-1	Fw:5′-TGGCTCTAGCTCTCACCCTTC-3′	*XM_003449328·3*
		Rv:5′-CTGAGCCAGGATGACCTTGAACT-3′	
*sglt1*	Sodium/glucose cotransporter 1	Fw:5′-CATCGCGTATCCGAAACTGG-3′	*XM_019361133·1*
		Rv:5′-CTCTGCCAGCAATCATGAGTT-3′	
*muc2*	Mucin-2	Fw:5′-AGAAAGGATGATGCAAGCCCT-3′	*XM_025902524·1*
		Rv:5′-ACGGCAAAGTGATACTCTGCATAT-3′	
Fatty acid biosynthesis genes			
*fads2*	Fatty acid desaturase 2	Fw:5′-CTGGTCATCGATCGAAAGGT-3′	*XM_005470633·4*
		Rv:5′-GCGGCTTCAGAAACTTATGC-3′	
*fads6*	Fatty acid desaturase 6	Fw:5′-GCGGGAAAGCTAAAGGTGTT-3′	*XM_025909732·1*
		Rv:5′-TGGCATCCTCTCCAGCATAG-3′	
*elovl5*	Elovl fatty acid elongase 5	Fw:5′-GATCACGTTCCTGCACCTCT-3′	*NM_001279460·1*
		Rv:5′-GCTGAGAGGCCATAGTACGA-3′	
Housekeeping genes			
*18 S*		Fw:5′-GGCAACCAACGGTAAAACAA-3′	DQ397879·1
		Rv:5′-AGCTAGCTGCGTTCTTCATTG-3′	

#### Analysis of the intestinal microbiota

##### 16S rDNA amplicon sequencing

The gut microbiota of all samples (midgut) was determined simultaneously for all samples using the following steps: DNA extraction, PCR, sequencing and post-sequencing analysis (see protocol description in ref. 31). As required, strict laboratory controls were performed to avoid contamination from PCR reagents and laboratory equipments.

##### Methods of 16S rRNA sequence analysis

Raw sequences were processed using the DADA2 (deficiency of adenosine deaminase 2) pipeline in R^(32)^. Forward and reverse reads were separately quality-trimmed and primer sequences removed. DADA2 was used to correct for sequencing errors, infer ‘amplicon sequencing variants’ (ASV) from both forward reads and reverse reads separately, and merge the forward and reverse ASV to construct the full target amplicon (based on an overlap of at least fifty nucleotides). DADA2 was used to remove chimeric ASV and also to assign taxonomy to the inferred ASV using the naïve Bayesian classifier method with the SILVA SSU rRNA database (version 138) as a training dataset^(33)^. From the raw sequences, 2037 ASV were inferred after processing with DADA2. The outputs of the DADA2 package were a FASTA file containing the DNA sequence of each ASV, a count table showing the abundance of each ASV in every sample and a taxonomic table showing the taxonomic classification of each ASV. The inferred ASV were aligned using MUSCLE, and a maximum likelihood tree was calculated in MEGA X^(34)^. The sequencing raw data was deposited in the NCBI (National Center for Biotechnology Information) Sequence Read Archive database (accession: PRJNA826326).

#### Digestibility test

A digestibility assay was carried out on the same fish used for the growth test, 3 weeks after feeding with the different diets. Chromium oxide (Sigma-Aldrich) was introduced into the fish diet as an inert marker at a level of 1 % (Cr_2_O_3_, 10 g/kg) relative to the previous formulation and fed to the ﬁsh under the same conditions as the growth experiment. The protocol for collecting faeces and determining chromium oxide concentrations in diets and faeces were described according to ref. 35 and reported in ref. 6.

### Statistical analyses

Mean values and standard deviation (s d, *n* 3) of the tanks were used as experimental units for statistical analyses. Data were tested for homogeneity and normality of the variances using the Shapiro–Wilk and Bartlett tests, respectively. The heterogeneous or non-normally distributed data and percentage values were log-transformed before analyses. A two-way ANOVA (using substrate for larval culture and chitinase dose as factors) was applied to the data collected (Table 8), followed by Dunnett’s test to compare each of the treatment means with the control means. In the statistical analysis tests used, *P* < 0·05 or *P* < 0·01 or *P* < 0·001 were considered statistically signiﬁcant. Significant differences between means showing effects of BSF larval production substrate (FO; VG) are indicated by capital letters A, B and C above the graphs, and effects of the dose of chitinase D0, D2 and D5 are also represented by capital letters A, B and C in front of the figure legends. When there is an interaction effect between BSF larval production substrate and the dose of chitinase in the diet, ANOVA1 has been performed and significant differences are represented by lower-case letters a, b and c above the bars of the graphs. Statistical analyses were performed using R 3.03 software.

Statistical analysis of the microbiota of each gut sample was performed in R using the phyloseq and microbiome R packages^(36)^. *α*-Diversity measures (Shannon diversity and Chao1 richness) were calculated from the untransformed ASV counts per sample. To test the effect of the fish diet on the *α*-diversity of the gut microbiome, a two-way ANOVA was performed on calculated *α* diversity measures using GraphPad Prism version 9.1 for Mac (GraphPad software).

The differences in microbiome composition (*β*-diversity) between the different gut samples were also tested. The weighted UniFrac distances were calculated using log-transformed abundance data (using the maximum likelihood tree obtained from all inferred ASV), and a principal coordinate analysis was used to plot the calculated *β*-diversity between the gut samples. To determine if the microbiome was statistically different between diet groups, we used the R package ‘vegan’. We tested for homogeneity of within-group dispersion (*betadisper* function), and a PERMANOVA (permutational multivariate analysis of variance) analysis was performed with the *adonis* function with diet as the variable.

The taxa contributing most to the observed differences in microbiomes between fish fed different diets were determined using the linear discriminant analysis (LDA) effect size (LEfSe) algorithm^(37)^. This algorithm determines differentially abundant taxa between gut samples from fish fed different diets using the non-parametric factorial Kruskal–Wallis rank-sum test (*α* = 0·05, all-against-all comparison for multi-class analysis) followed by a LDA to estimate the effect size of each differentially abundant taxon. From this dataset, a cladogram was created that indicates the taxonomy of each differentially abundant feature. Furthermore, the Log10 LDA scores of the differentially abundant taxa for each diet group were calculated and plotted.

Additionally, the relative abundances of phyla and families were calculated with the microbiome R package. The threshold for ASV was 0·5 % relative abundance and present in 50 % of the samples.

## Results

### Growth performance, feed utilisation and nutrient digestibility

**Fig. 1.**
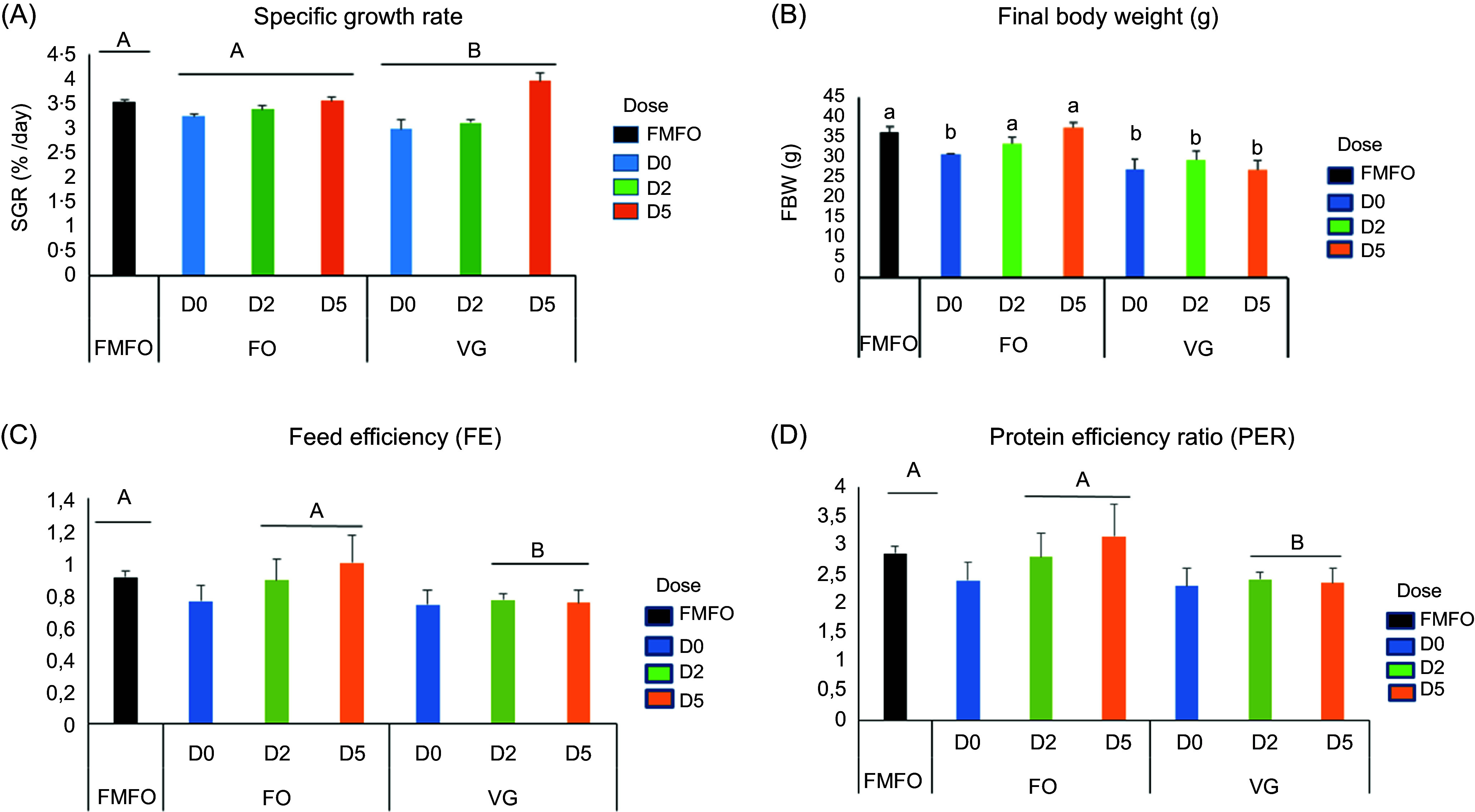
Growth performance and feed utilisation of juvenile Nile tilapia fed different experimental diets for 53 d. (a) Specific growth rate (SGR), (b) final body weight (FBW), (c) feed efficiency and (d) protein efficiency ratio. Data are expressed as mean ± sd, *n* 3. Significant differences between means are represented by letters indicating an overall effect of substrate production in black soldier fly larvae FO; VG ((a), (b), (c) above the graph), or a dose effect of chitinase D0, D2 and D5 ((a), (b), (c) in front of the legend) or an interaction effect of substrate and chitinase dose ((a), (b), (c) above the bars) (*P* < 0·05). FMFO, control diet with fishmeal and fish oil.

Figure 1 shows feed utilisation and growth data for fish fed BSF larval meal with or without chitinase. After 53 d of feeding, the BSF/FOS diets induced comparable growth (SGR) to the FMFO control diet, but better than the BSF/VGS diets (*P* < 0·05) (Fig. 1(a)). Similar to growth, the FE and PER were higher in fish fed BSF/FOS diets than in those fed BSF/VGS diets (*P* < 0·05) (Fig. 1(c) and (d)). The dietary chitinase had no effect on growth or feed utilisation in fish. However, there was no significant difference in FI and survival rate (82·2–93·3 %) of the different diets (Table 5).

**Table 5.** Growth performance, feed utilisation of juvenile Nile tilapia fed different experimental diets for 53 d

Parameters	FMFO		FOD0		FOD2		FOD5		VGD0		VGD2		VGD5	
	Mean	sd	Mean	sd	Mean	sd	Mean	sd	Mean	sd	Mean	sd	Mean	sd
Survival (%)	88·88	1·92	82·22	5·09	93·33	8·82	91·11	1·92	84·44	6·94	90·00	5·77	85·55	1·92
Initial body weight (g)	5·59	0·02	5·50	0·08	5·54	0·02	5·65	0·04	5·54	0·06	5·61	0·02	5·58	0·02
Feed intake (FI, g/fish/d)	0·63	0·05	0·63	0·08	0·59	0·06	0·61	0·09	0·55	0·03	0·58	0·06	0·53	0·02

FMFO, control diet with fishmeal (FM) and fish oil (FO); FOD, diet based on black soldier fly larvae meal obtained from larvae grown on fish offal substrate; VGD, diet based on black soldier fly larvae meal grown on a vegetable culture substrate.

The number added to the name of the diet indicates the dose of chitinase (2 or 5 g/kg of feed).

Mean ± sd, *n* 3.

**Fig. 2.**
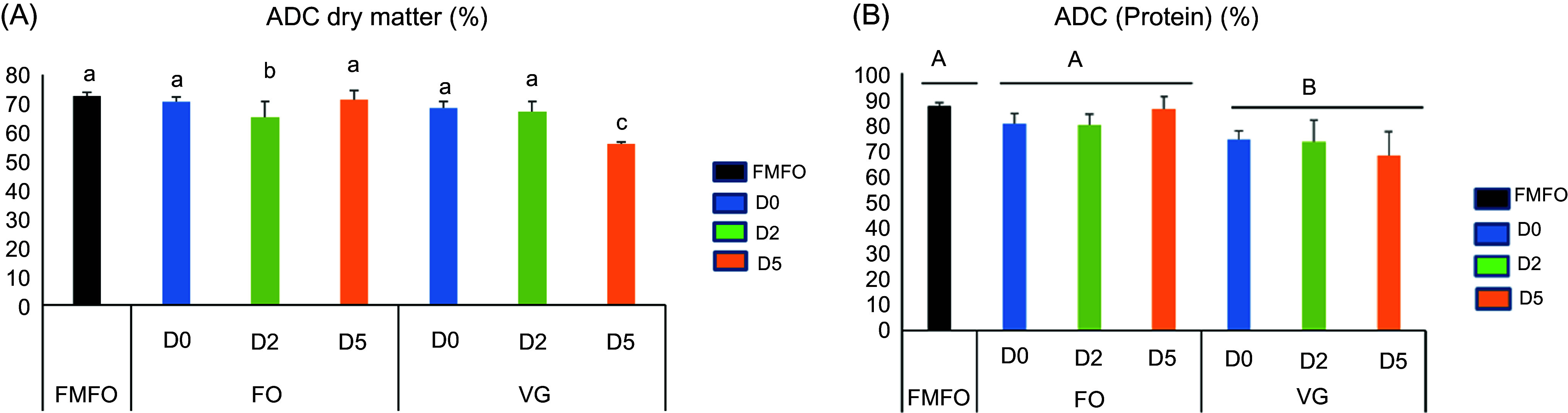
Apparent digestibility coefficients (ADC) of nutrients in juvenile Nile tilapia fed the different experimental diets for 53 d. (a) ADC DM and (b) ADC protein. Data are expressed as mean ± sd, *n* 3. Significant differences between means are represented by letters indicating an overall effect of substrate production in black soldier fly larvae FO; VG ((a), (b), (c) above the graph), or a dose effect of chitinase D0, D2 and D5 ((a), (b), (c) in front of the legend) or an interaction effect of substrate and chitinase dose ((a), (b), (c) above the bars) (*P* < 0·05). FMFO, control diet with fishmeal and fish oil.

Apparent digestibility coefficient of dry matter (ADC_DM_) values were high and comparable between fish receiving the FMFO control diet and fish receiving the different BSF meal diets, except for a decrease induced by the VGD5 and FOD2 diet (*P* < 0·05) (Fig. 2(a)). Similar to growth and feed utilisation, the BSF/FOS led to higher apparent digestibility coefficient of protein (ADCprotein) values than the BSF/VGS. Thus, the ADCprotein of fish fed the BSF/FOS diets was comparable to that of fish fed the FMFO control diet (*P* < 0·05) (Fig. 2(b)). The addition of chitinase to the diets did not affect fish protein digestibility. The apparent digestibility coefficient of lipid (ADC lipid) values were high and did not differ between fish fed the different diets (Table 6).

**Table 6.** Digestive enzyme activities and apparent digestibility coefficients of lipid (ADC lipid) of juvenile Nile tilapia fed experimental diets for 53 d

Parameters	FMFO		FOD0		FOD2		FOD5		VGD0		VGD2		VGD5	
	Mean	sd	Mean	sd	Mean	sd	Mean	sd	Mean	sd	Mean	sd	Mean	sd
Intestine alkaline phosphatase activity (mU mg.protein^−1^)	35·97	13·41	42·32	2·52	31·69	8·19	30·41	11·27	46·46	5·73	39·20	9·0	32·55	1·79
Stomach chitinase activity (mU mg.protein^−1^)	3·94	1·95	4·67	1·68	3·24	2·22	2·69	0·93	5·60	1·52	2·87	1·01	4·88	1·64
ADC (ipid) (%)	95·75	0·23	88·72	1·73	90·26	4·93	89·52	2·56	91·69	2·98	92·21	3·78	90·31	7·50

ADC, apparent digestibility coefficient; FMFO, control diet with fishmeal (FM) and fish oil (FO); FOD, diet based on black soldier fly larvae meal obtained from larvae grown on fish offal substrate; VGD, diet based on black soldier fly larvae meal grown on a vegetable culture substrate.

The number added to the name of the diet indicates the dose of chitinase (2 or 5 g/kg of feed).

Mean ± sd, *n* 3.

### Activities of digestive enzymes

**Fig. 3.**
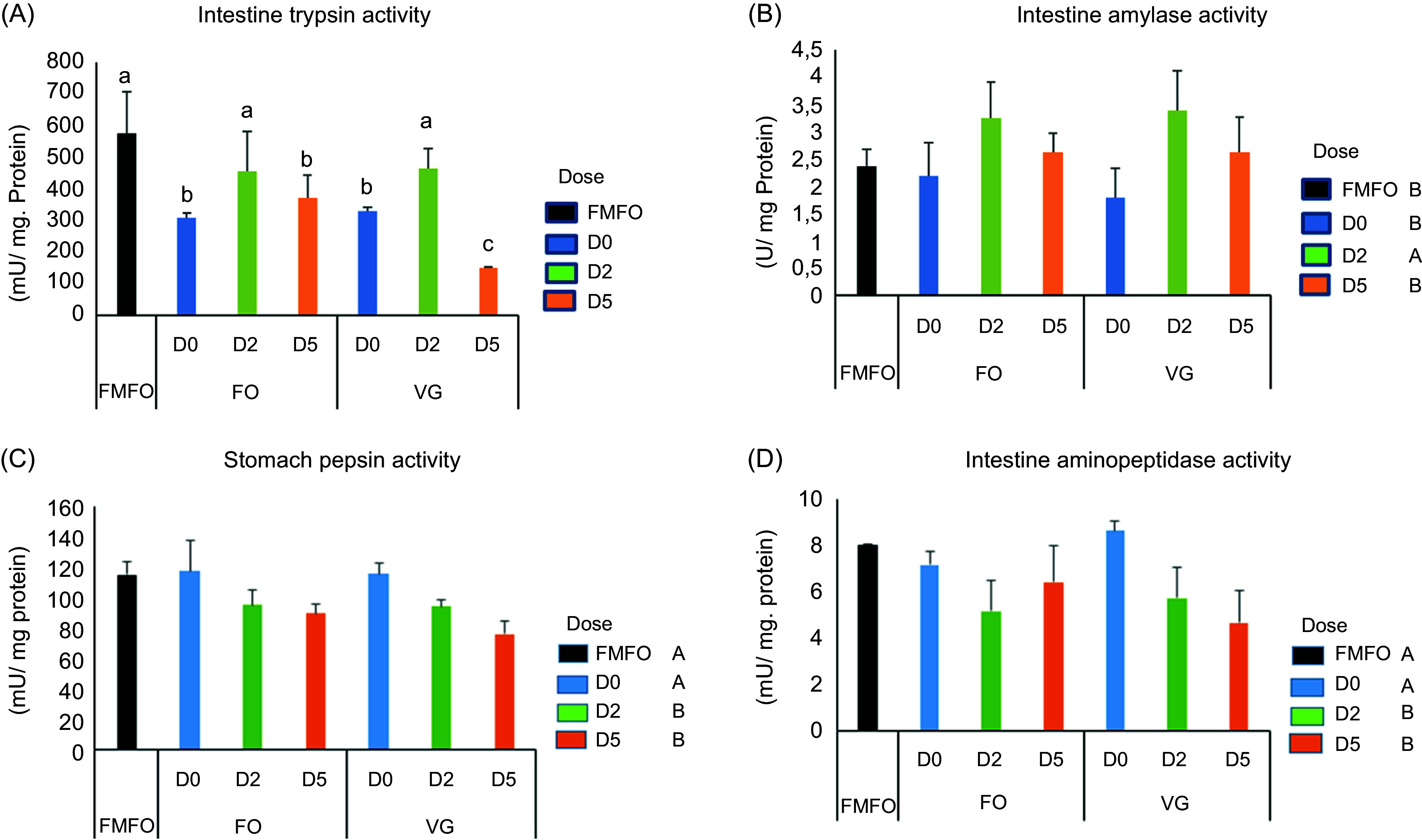
Digestive enzyme activities of juvenile Nile tilapia fed experimental diets for 53 d. (a) Intestine trypsin activity, (b) intestine amylase activity, (c) stomach pepsin activity and (d) intestine aminopeptidase activity. Data are expressed as mean ± sd, *n* 3. Significant differences between means are represented by letters indicating an overall effect of substrate production in black soldier fly larvae FO; VG ((a), (b), (c) above the graph), or a dose effect of chitinase D0, D2 and D5 ((a), (b), (c) in front of the legend) or an interaction effect of substrate and chitinase dose ((a), (b), (c) above the bars) (*P* < 0·05). FMFO, control diet with fishmeal and fish oil.

The activities of the digestive enzymes are shown in Fig. 3(a)–(d) and Table 6. The BSF meal did not affect the activity level of the digestive enzymes tested regardless of the substrate, except for a reduction in the trypsin activity level (Fig. 3(a), *P* < 0·001). The low dose of dietary chitinase prevented this reduction, but not the high dose.

Therefore, trypsin values were significantly higher in fish fed the FMFO control diet than in those fed the VGD0, FOD0, VGD5 and FOD5 diets, but comparable between those fed the FMFO control diet and those fed the low chitinase dose diets VGD2 and FOD2. Therefore, trypsin values were significantly higher in fish fed the FMFO control diet than in those fed the VGD0, FOD0, VGD5 and FOD5 diets, but comparable between those fed the FMFO control diet and those fed the low chitinase dose diets VGD2 and FOD2. The low dose of dietary chitinase in the diets also increased amylase activity in the gut of fish compared with those fed the diets without chitinase (*P* < 0·01) (Fig. 3(b)). The addition of the high dose of chitinase reduced the activity level of some digestive enzymes. Pepsin activity was significantly higher in fish fed without chitinase (VGD0 or FOD0) than in those fed with dietary chitinase (*P* < 0·001) but was comparable between those fed without chitinase and the FMFO control diet (Fig. 3(c)). Therefore, the addition of chitinase to the FMFO diets reduced pepsin activity in the fish stomach (Fig. 1(c)). Similarly, aminopeptidase activity in the gut of fish fed BSF without chitinase was comparable to those fed the FMFO control diet, but the addition of chitinase to BSF-based diets reduced aminopeptidase activity in fish (*P* < 0·001) (Fig. 3(d)). Values for alkaline phosphatase activity in the intestine and chitinase activity in the stomach were not affected by either the type of BSF meal or the dietary chitinase dose (Table 6).

### Relative expression of enzymatic and lipid metabolism genes

**Fig. 4.**
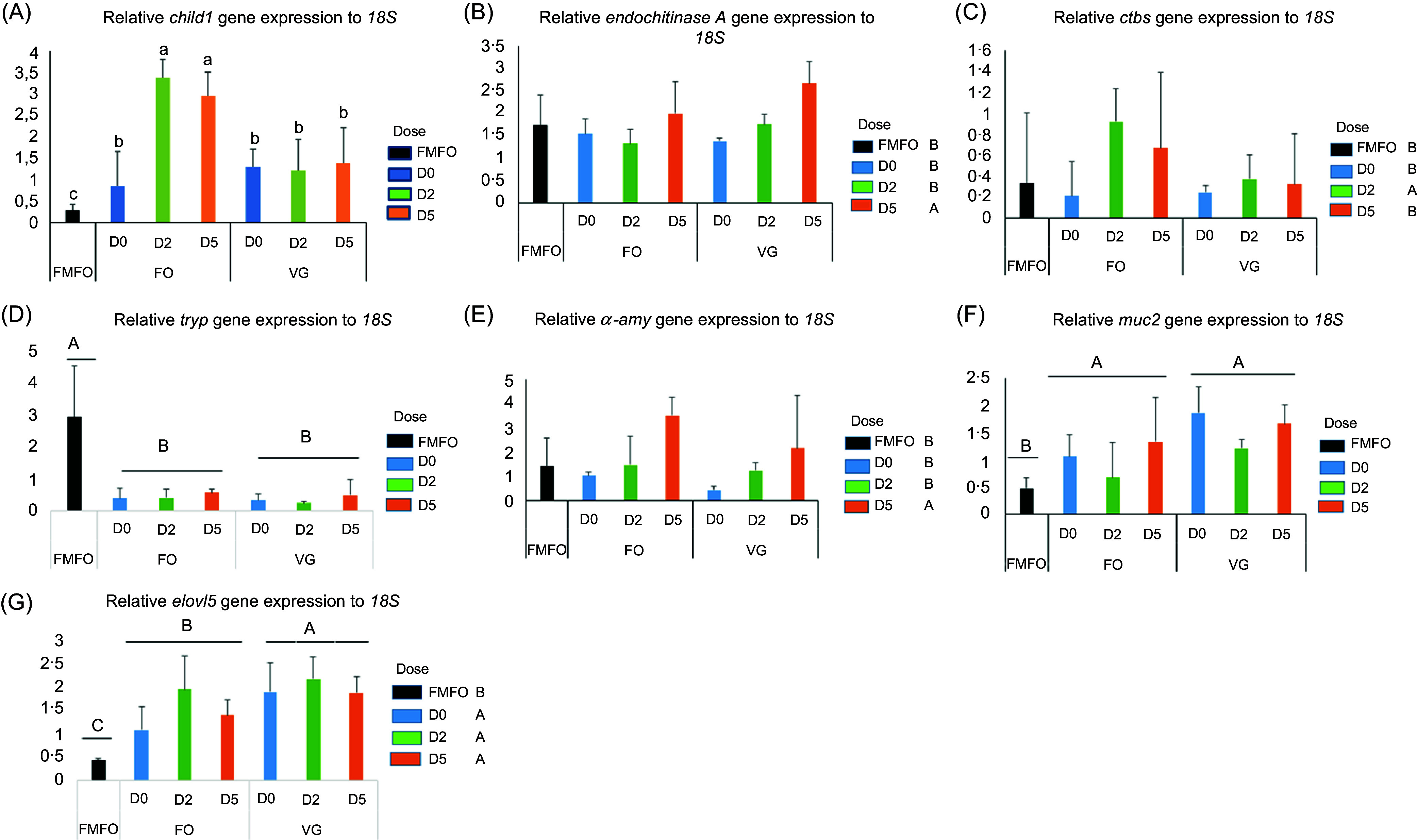
The relative expression levels of enzymatic genes (a) *chid1*, (b) *endochitinase A*, (c) *ctbs*, (d) *tryp* (e) *α*-*amy*, (f) *muc2* and (g) *elovl5* of Nile tilapia juveniles fed experimental diets for 53 d. Data are expressed as mean ± sd, *n* 3. Significant differences between means are represented by letters indicating an overall effect of substrate production in black soldier fly larvae FO; VG ((a), (b), (c) above the graph), or a dose effect of chitinase D0, D2 and D5 ((a), (b), (c) in front of the legend) or an interaction effect of substrate and chitinase dose ((a), (b), (c) above the bars) (*P* < 0·05). FMFO, control diet with fishmeal and fish oil.

The BSF meal in the fish feed increased *chid1* gene expression compared with the FMFO control diet (Fig. 4(a), *P* < 0·01). The combination of dietary chitinase with BSF/FOS increased *chid1* gene expression in fish fed the FOD2 and FOD5 diets compared with those fed without dietary chitinase (FOD0). Only the high dose of chitinase in the BSF-based diets increased the relative expression of the *endochitinase A* gene and the *α*-*amy* gene in fish compared with the other diets (*P* < 0·05, Fig. 4(b) and (e)). On the other hand, it was the low dose of chitinase in the BSF-based diets that increased *ctbs* gene expression in fish compared with the other diets (*P* < 0·05, Fig. 4(c)). A significant decrease in *tryp* gene expression was observed in BSF-fed fish compared with the FMFO control (*P* < 0·001, Fig. 4(d)). In contrast, an increase in muc2 gene expression was observed in fish fed BSF meal-based diets compared with the FMFO control (*P* < 0·01, Fig. 4(f)). The relative expressions of *pept2*, *apoa1*and *sglt1* genes were not affected by either BSF type or dietary chitinase (Table 7). An increase in relative *elovl5* gene expression was observed in fish fed BSF-based diets compared with the FMFO control, and this increase was more accentuated in fish fed BSF/VGS than BSF/FOS (*P* < 0·05) (Fig. 4(g)). The relative expressions of other lipid metabolism genes such as *fads2* and *fads6* were not affected by either the BSF type or dietary chitinase (Table 7).

**Table 7.** Relative expression levels to 18S of enzymatic genes and genes involved in FA biosynthesis in juvenile Nile tilapia fed experimental diets for 53 d

Parameters	FMFO		FOD0		FOD2		FOD5		VGD0		VGD2		VGD5	
	Mean	sd	Mean	sd	Mean	sd	Mean	sd	Mean	sd	Mean	sd	Mean	sd
*pept2*	1·54	0·65	0·85	0·27	1·13	0·63	0·51	0·28	0·91	0·60	1·09	0·49	0·21	0·13
*sglt1*	1·43	1·03	1·06	0·66	1·45	0·56	2·13	1·24	1·93	1·13	2·41	1·16	1·00	0·38
*apoa1*	1·06	0·45	1·05	1·01	0·62	0·65	1·00	0·75	1·52	1·41	1·42	1·06	1·79	0·07
*fads2*	0·86	0·10	2·08	1·39	1·20	0·81	1·69	0·91	2·31	1·02	1·89	0·71	0·99	0·63
*fads6*	0·70	0·5	0·88	0·70	0·71	0·48	0·63	0·60	1·48	0·51	0·76	0·20	0·76	0·17

FA, fatty acid; FMFO, control diet with fishmeal (FM) and fish oil (FO); FOD, diet based on black soldier fly larvae meal obtained from larvae grown on fish offal substrate; VGD, diet based on black soldier fly larvae meal grown on a vegetable culture substrate.

The number added to the name of the diet indicates the dose of chitinase (2 or 5 g/kg of feed).

Mean ± sd, *n* 3.

### Diversity and taxonomic composition of the gut microbiota

**Fig. 5.**
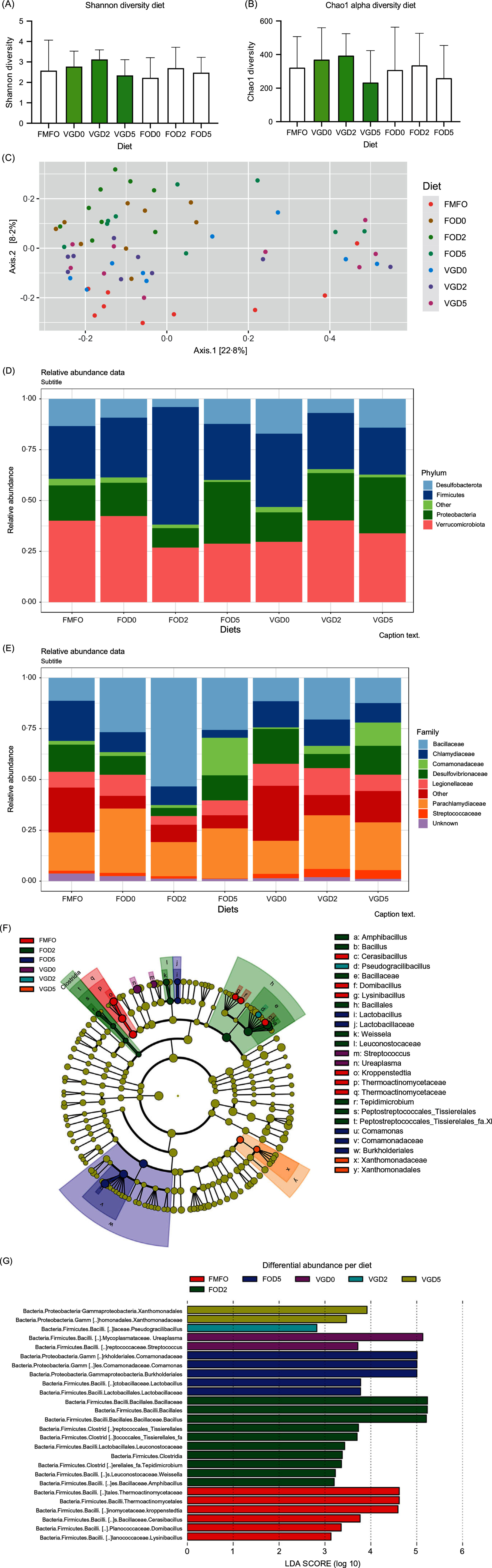
The gut microbiota of juvenile Nile tilapia fed with fishmeal (FM) and fish oil (FO) or black soldier fly (BSF) meals enriched or not with long-chain (LC)-PUFA and supplemented with chitinase for 53 d: (a) *α*-Diversity indices (Shannon index) and (b) Chao1 *α* diversity of bacterial communities, and (c) the *β*-diversity (based on Bray–Curtis dissimilarity ordination). (d) The average relative abundance represented at the phylum level and (e) the average relative abundance represented at the family level. (f) Cladogram showing significantly abundant taxonomic groups identified based on linear discriminant analysis effect size (LEfSe) analysis (*P* < 0·05). ASV are coloured according to their taxonomic classification, and amplicon sequencing variants (ASV) without any assignment are shown in yellow. (g) LEfSe analysis shows distinctive genera of bacteria for each diet (mean ± sd, *n* 9). FMFO, control diet with fishmeal and fish oil; FOD, diet based on black soldier fly larvae meal obtained from larvae grown on fish offal substrate; VGD, diet based on black soldier fly larvae meal grown on a vegetable culture substrate.

The *α* diversity indices (Shannon and Chao1 index, indicating the number of microbial species per sample) of the gut microbiota communities were not affected by either BSF type or dietary chitinase (Fig. 5(a) and (b)). The *β*-diversity (based on Bray–Curtis dissimilarity ordination) showed that microbiome composition was significantly affected by BSF type (*P* = 0·001; R2 = 0·09) and chitinase dose (*P* = 0·045; R2 = 0·04) (Fig. 5(c)). The phyla *Verrucomicrobiota* (*Parachlamydiaceae*, *Chlamydiaceae*), *Firmicutes* (*Bacillaceae*, *Streptococcaceae*), *Proteobacteria* (*Comamonadaceae*, *Legionellaceae*) and *Desulfobacterota* (*Desulfovibrionaceae*) were identified in all fish groups (Fig. 5(a) and (b)).

The BSF/FOS associated with dietary chitinase increased the relative abundance of *Firmicutes* (*Amphibacillus*, *Bacillus* and *Tepidimicrobium*) in fish fed the FOD2 diet compared with all other diets (Fig. 5(d)). Moreover, the FOD2 diet decreased *Proteobacteria* in these fish compared with all other diets (Fig. 5(d)). The fish fed the diet without chitinase FOD0 did not have distinctive bacterial genera compared with the other diets (Fig. 5(f)). The BSF/VGS increased the differential abundance per diet of *Firmicutes* (*Streptococcus*, *Ureaplasma*) in fish fed the VGD0 diet, *Firmicutes* (*Pseudogracilibacillus*) in fish fed the VGD2 diet and *Proteobacteria* (*Xanthomonadaceae*) in those fed the VGD5 diet compared with the other diets (Fig. 5(f) and (g)). The FMFO control diet increased the differential abundance per diet of some *Firmicutes* (*Cerasibacillus*, *Domibacillus*, *Lysinibacillus* and *Kroppenstedtia*) in fish compared with the other diets (Fig. 5(f) and (g)).

**Table 8.** *P*-values of the two factors dose of chitinase (D) and substrate for BSF larvae production (sub) and their interaction (dose × sub)

Variables	Experimental factor		
	Dose	Sub	Dose × Sub
Growth performance and feed utilisation			
Survival (%)	0·05	0·644	0·445
Initial body weight (g)	0·073	0·807	0·118
Final body weight (g)	0·59	< 0·001	0·009
Specific growth rate (SGR, % d^−1^)	0·958	< 0·001	0·056
Feed intake (FI, g/fish/d)	0·874	0·101	0·589
Feed efficiency (FE)	0·42	< 0·01	0·21
Protein efficiency ratio (PER)	0·42	< 0·01	0·21
Apparent digestibility coefficients (ADC)			
ADC(DM) (%)	0·002	0·001	0·0004
ADC(Protein) (%)	0·305	0·002	0·173
ADC(Lipid) (%)	0·612	0·127	0·897
Digestive enzyme activities			
Amylase activity	0·004	0·628	0·687
Pepsin activity	< 0·001	0·563	0·542
Trypsin activity	0·001	< 0·001	0·03
Aminopeptidase activity	< 0·001	0·975	0·06
Alkaline phosphatase activity	0·101	0·224	0·857
Chitinase activity	0·174	0·316	0·418
Expression of enzymatic genes			
* endochinase A*	0·02	0·17	0·289
* ctbs*	0·03	0·04	0·109
* chid1*	0·001	0·009	0·03
* pept2*	0·019	0·174	0·795
* tryp*	0·05	0·0002	0·99
* a*-*amy*	0·02	0·278	0·679
* sglt1*	0·642	0·868	0·127
* apoa1*	0·766	0·266	0·937
* muc2*	0·171	0·008	0·71
Expression of lipid metabolism genes			
* fads2*	0·677	0·133	0·399
* fads6*	0·395	0·239	0·584
* elovl5*	0·002	0·042	0·588

BSF, black soldier fly.

When *P* < 0·05, the statistical differences between regimens for each factor are placed in the tables and figures showing the parameters concerned.

## Discussion

### Effects of enrichment of black soldier fly with long-chain PUFA and/or dietary chitinase on growth, feed utilisation and digestive features

All groups of fish had a high survival rate, and the type of larval rearing substrate or the dose of chitinase added to the diet did not affect the survival. After 53 d of feeding, the BSF/FOS-based diets induced better FE, PER and growth than the BSF/VGS-based ones, indicating a better stimulation of growth processes by LC-PUFA enrichment compared with PUFA alone. In salmonids such as rainbow trout, a DHA-enriched diet has been reported to improve juvenile growth performance compared with an LA-enriched diet^(38)^. To explain this, these authors hypothesised that the intestinal membranes of fish fed the DHA-enriched diet were more flexible and fluid and their thickness was reduced compared with those fed the LA-enriched diet. Similarly, in our study, the membranes of tilapia fed a diet enriched with LC-PUFA could be thinner, more flexible and more fluid than those of fish fed an ALA-enriched diet. The significant decrease in growth of fish fed BSF/VGS-based diets compared with BSF/FOS-based diets could be explained by the lack of EPA + DHA and the high composition of saturated, MUFA and LA + ALA in the BSF/VGS-based diets. It has already been reported that lipids in fish feed can be used to spare proteins in order to maximise their use for growth and improve the PER^(39)^. In our study, a balanced diet containing PUFA and LC-PUFA might have stimulated the protein-sparing process and thus overall dietary feed utilisation in fish compared with diets containing only PUFA. The LC-PUFA contained in the BSF/FOS meal induced a better digestibility of the proteins in these diets and consequently improved feed utilisation and growth of these fish.

Chitinase in different BSF diets would have hydrolysed the *β*-1,4 glycosidic linkages between the constituent monosaccharid residues present in the chitin polysaccharide chains into trimers, dimers and monomers of N-acetylglucosamine^(40)^. The release of these monosaccharides from the chitin chain would increase intestinal amylase activity in fish fed BSF-based diets containing the low-dose chitinase compared with diets without chitinase.

The addition of chitinase to BSF-based diets decreased pepsin and aminopeptidase activities in fish regardless of the type of BSF meal. The products of chitin decomposition by chitinase such as chitosan, can, at high levels, inhibit pepsin activity. Similar results were obtained when pepsin solutions from porcine gastric mucosa were added to buffers with different concentrations of chitosan from shrimp shells and a concentration of chitosan > 10 g/l inhibited the activity of pepsin *in vitro* ^(41)^.

In the present study, chitinase was observed to be active in the stomach of tilapia, although there was no significant difference between the different diets. A previous study revealed chitinase activity in the digestive tract of *O. niloticus* ^(42)^. Chitinase facilitates the enzymatic breakdown of chitin into its monomer GlcNac, which is then absorbed and used as an energy source^(43)^. For complete degradation, the chitinolytic system must be complete, that is, sufficient endochitinase and exochitinase must be present^(42)^. The breakdown of chitinase occurs in two consecutive steps: first, the hydrolysis into oligomers (mainly dimers) by endochitinase and then the degradation of these oligomers into free N-acetylglucosamine by exochitinase^(42)^.

### Influence of the enrichment of black soldier fly and dietary chitinase on the relative expression of enzymatic and lipid metabolism genes

In all diets, the BSF meal increased *chid1* gene expression in fish compared with the FMFO control diet, and the combination of dietary chitinase with BSF/FOS increased *chid1* gene expression in fish fed the FOD2 and FOD5 diets compared with those fed without dietary chitinase (FOD0). Although chitinolytic activity did not differ in the stomachs of fish fed the different diets, dietary chitinase increased *ctbs* and *endochitinase.* Gene expression in fish fed the BSF meal-based diets allows these fish to better digest chitin. Other studies have shown that replacing 50 % of FM with BSF meal in the diet of zebrafish (*Danio rerio*) for 6 months increased the expression of chitinase genes *chia.3* and *chia.5* in fish compared with the control without BSF^(44)^. A significant decrease in *tryp* gene expression was observed in fish fed BSF compared with the FMFO control. The decrease in expression of this gene could explain the decrease in trypsin activity in the intestine of these fish. In this study, a trend of increased *muc2* gene was observed in fish fed the BSF-based diets and especially in those fed the VGD0 diet. BSF/VGS had increased the number of goblet cells in the proximal intestine of the fish (30). This result is consistent with previous studies that reported that BSF supplementation in the diet of barramundi (*Lates calcarifer*) improved the barrier functions of the skin mucosa; an increase in acid mucins can produce mucus that facilitates many biological activities to prevent colonisation of infectious micro-organisms^(45)^. The relative expressions of *apoa1* and *sglt1* genes were not affected by the type of BSF or dietary chitinase. These results showed that BSF and chitinase in the fish diet did not interfere with the production of apo A-1 and sodium/glucose cotransporter 1.

An increase in relative *elovl5* gene expression was observed in fish fed BSF-based diets compared with the FMFO control, and this increase was more accentuated in fish fed BSF/VGS than BSF/FOS. The *n*-3/*n*-6 ratio was higher in the FMFO control diet than in the other BSF-based diets. Moreover, this ratio was lower in the BSF/VGD diets compared with the BSF/FOD diets and the FMFO control. Fish consuming this diet need to elongate more carbon chains to produce enough LC-PUFA from PUFA, which could explain the high expression of the *elovl5* gene in fish fed the VGD2 diet. This confirms our previous studies that showed that Nile tilapia juveniles fed a BSF/VGD diet had increased *elovl5* expression^(6)^.

### Influence of the enrichment of black soldier fly and dietary chitinase on the gut microbiota

In this study, the phylum *Verrucomicrobiota*, *Firmicutes* and *Proteobacteria* were identified in all fish. The gut microbiota of different fish species often consists to 94 % of the phyla *Actinobacteriota*, *Bacteroidota*, *Firmicutes* and *Proteobacteria* regardless of diet^(46)^. *Verrucomicrobiota* in the fish gut would come from the water^(47)^ because the fish are constantly drinking tank water. The *α* diversity indices (Shannon and Chao1 indices, indicating the number of microbial ASV per sample) of the gut microbiota communities were not different between the different diets. Fish were reared in an aquaculture recirculation system, which could explain the lack of difference in ASV richness. In addition, feeds are prepared from the same ingredients except for BSF meals. The gut microbiota of fish could partly be derived from the diet, but the specific composition of the diet could also modulate the microbiome^(15)^. In teleost fish, approximately 90 % of the gut microbiota is composed of *Bacteroidetes, Firmicutes* and *Proteobacteria* ^(48)^. The most abundant phyla found in Nile tilapia were *Actinobacteria*, *Bacteroidetes*, *Cyanobacteria*, *Firmicutes*, *Fusobacteria* and *Proteobacteria* ^(49)^. BSF/FOS combined with the low dose of dietary chitinase increased the relative abundance of multiple *Firmicutes* species and decreased *Proteobacteria* in fish fed the FOD2 diet compared with any other diet. *Proteobacteria* include several bacterial species that are potentially pathogenic to fish^(50)^, and the phylum *Firmicutes* contains bacteria that are beneficial to fish^(51)^. When rainbow trout were fed BSF meal, an increase in bacteria such as *Acinetobacter*, *Actinobacteriota*, *Actinomyces*, *Bacillaceae*, *Brevibacterium*, *Corynebacterium*, *Firmicutes*, *Lactobacillales*, *Oceanobacillus*, *Staphylococcus* and a decrease in *Proteobacteria* was observed in the gut microbiota of the fish compared with those fed the control diet^(52)^. The *Firmicutes* such as *Lactobacillus*, which was abundant in fish fed the FOD5 diet, are beneficial gut microbes because of their ability to improve digestibility, mucosal tolerance, immune response and disease resistance in the host^(53)^. These bacteria are known for the production of bactericidal compounds and lactic acid which can prevent the colonisation of pathogens on the surface of the gut^(53)^ and even prevent or repair gut damage caused by anti-nutritional factors contained in vegetable-based ingredients like soyabean meal in fish diet^(54)^. The presence of *Lactobacillus* in fish fed the FOD5 diet could also explain the beneficial effects of this diet on digestibility, growth, intestinal health and better innate immune defence of these fish after being exposed to *E. coli* lipopolysaccharide^(30)^. Moreover, many species of bacteria belonging to the *Bacillaceae* family can produce chitinase^(55)^. In this study BSF/FOS combined with a low dose of chitinase promoted the abundance of *Bacillus* in fish fed the FOD2 diet. Furthermore, previous studies in Atlantic salmon and Atlantic cod (*Gadus morhua L*.) have shown that BSF larval meal can act as a substrate and selectively promote the multiplication of certain chitinolytic bacteria such as *Lactobacillaceae* and *Bacillaceae* in the fish gut^(15)^.

In the present study, LEfSe analysis showed more of the distinct bacterial genera of the phylum *Firmicutes* in fish fed BSF/FOS combined with chitinase (FOD2 and FOD5) than those fed the BSF/VGS-based diets (Fig. 5(g)). The presence of a greater number of distinct bacterial genera of the phylum *Firmicutes* in these fish could explain the better intestinal health of fish fed FOD5 compared with those fed VGD0 or VGD5^(30)^. It is thought that species-rich communities may provide additional metabolic capabilities to the host^(56)^ and compete with pathogens for nutrients and colonisation^(57)^. For example, to reduce pathogen adhesion, *Lactobacillus* can create a biofilm in the gut^(58)^. In the present study, fish fed the FOD2 diet had more distinctive genera of bacteria than the FMFO control. The *n*-3/*n*-6 ratio was 0·5 in the FMFO diet, 0·25 in the FOD2 diet and 0·17 in the BSF/VGS diets. Distinctive genera in the FOD2 diet included the genus *Weissella*, which have been reported among FA bioconversion bacteria^(59)^. Previous studies have reported that changes in the *n*-3 PUFA content of the diet have altered the *β*-diversity (i.e. microbial composition) of gut bacteria. Indeed, an increase in the ratio of *Firmicutes: Proteobacteria* was observed, including the genera *Leuconostoc*, *Streptococcus* and *Weissella* in the fish gut when the FO was replaced by the vegetable oil in the feed^(60)^. The low levels of *n*-3 FA in the diet of fish can be compensated by an increase in the abundance of FA-producing bacteria in the gut^(59)^. Our study does not seem to confirm this compensation for low levels of *n*-3 FA because FA-producing bacteria were not abundant in fish fed with ALA-enriched BSF/VGS compared with those fed with ALA + EPA-enriched BSF/FOD. The fact that BSF/VGS-based diets with the lowest *n*-3/*n*-6 ratio and lacking EPA and DHA have a low relative abundance of bacteria could suggest that there should be a minimum amount of LC-PUFA in the diet to promote the abundance of FA-producing bacteria.

Regarding other possible questions related to this study, we recall that results on intestinal histopathology and immune responses before and after an *Escherichia coli* lipopolysaccharide challenge have been reported elsewhere^(30)^.

In summary, the results of the present study showed that chitinase supplementation to a BSF-based diet enriched with LC-PUFA improved PER and digestibility, FE, and growth in Nile tilapia. The BSF/VGS and BSF/FOS meals associated or not with dietary chitinase differentially modulated the gut microbial composition of fish. The BSF/FOS meal associated with low-dose chitinase decreased the abundance of *Proteobacteria* in the fish gut, some of which are responsible for diseases, and increased the abundance of *Firmicutes*, some of which are responsible for FA bioconversion and others for chitin digestion. Overall, the results showed that BSF meal enriched with LC-PUFA and optimal dose of chitinase could be used in aquaculture feeds as an effective alternative to FM.
